# Ni Porous Preforms Compacted with Al_2_O_3_ Particles and Al Binding Agent

**DOI:** 10.3390/ma16030988

**Published:** 2023-01-20

**Authors:** Andrej Opálek, Peter Švec, Matúš Žemlička, Matej Štěpánek, Pavol Štefánik, Stanislav Kúdela, Naďa Beronská, Karol Iždinský

**Affiliations:** 1 Institute of Materials and Machine Mechanics, Slovak Academy of Sciences, Dúbravská Cesta 9, 845 13 Bratislava, Slovakia; 2 Institute of Physics, Slovak Academy of Sciences, Dúbravská Cesta 9, 845 11 Bratislava, Slovakia; 3 Institute of Construction and Architecture, Slovak Academy of Sciences, Dúbravská Cesta 9, 845 03 Bratislava, Slovakia; 4 Centre of Excellence for Advanced Materials Application, Slovak Academy of Sciences, Dúbravska Cesta 9, 845 11 Bratislava, Slovakia

**Keywords:** Ni-Al intermetallic, Al_2_O_3_ particles, metal-ceramic composite materials, porous compacts, oxidation, open porosity

## Abstract

This work presents an energy-efficient, cheap, and rapid production method of a metal–ceramic preform with open porosity suitable for liquid metal infiltration and filtration applications. It is based on cold isostatic pressing of a mixture of relatively hard Ni and Al_2_O_3_ powders with the addition of small amount of Al powders, acting as a binding agent. Open porosity is primarily controlled by Al_2_O_3_ particles partially separating Ni particles from mutual contacts. Cold isostatic pressed green compacts were subjected to thermal oxidation by heating in air to 600 °C, 700 °C, and 800 °C. The weight gain and open porosity of oxidized compacts were examined. The chemical composition and microstructure were analyzed by SEM-EDS and XRD techniques. The stability of preforms and the effect of thermal cycling on the open porosity were tested by thermal cycling in an inert Ar atmosphere in the temperature range up to 800 °C. It appeared that, in addition to NiO being an expected product of oxidation, Ni aluminides and spinel particles also played an important role in inter-particle bonding formation. Ni-NiO porous composites resist chemical corrosion and exhibit structural and chemical stability at higher temperatures and admixed Al_2_O_3_ particles do not deteriorate them. After subsequent infiltration with Al, it can offer a lower density than other materials, which could result in lower energy consumption, which is highly needed in industries such as the automotive industry.

## 1. Introduction

The porous medium can be defined as a material composed of a solid matrix consisting of interconnected cavities. Porous metals and metal foams (closed-porous materials) have a combination of properties that cannot be obtained with dense polymers. For example, metallic porous materials’ mechanical strength, stiffness, and energy absorption are much higher than the absorption of polymeric foams [1,2]. They are thermally and electrically conductive and retain their mechanical properties at higher temperatures. The sintered powder has been successfully used, e.g., for fabricating filters, batteries, self-lubricating bearings, etc. These materials are still used in high-volume applications. Generally, the choice of material structure is based on the final application. Foams with closed pores provide good mechanical properties but do not allow access to the internal volume of the material. Therefore, they are mainly used for construction applications. In contrast, the open porosity is important when bonding with the material’s interior is required, such as in filter elements. Open-porous materials can also be used as skeletons for infiltration by molten metal [2,3].

Recently, there is a growing demand for metallic porous materials in metallurgy, new catalysts, electronic equipment, aerospace, biomedicine, and environmental protection; therefore, higher requirements for their overall porosity and pore structure properties such as morphological homogeneity have been proposed [4,5].

The porous skeleton can be stabilized by adding ceramic particles to the molten metal, which adhere to the gas/metal interfaces during foaming and prevent coalescence of the pores. One of the foaming processes (“Alcan process”) [6] utilizes liquid metal matrix composites (MMC) containing 10–20 vol.% of particles (typically SiC or Al_2_O_3_ particles of 10 μm diameter), into which the gas is injected. Using this approach, it is possible to produce high-porous metallic materials. However, the high content of ceramic particles makes the product very brittle and difficult to machine. In addition, the conventional fabrication of metal, ceramic or metal–ceramic porous skeletons is technologically, energetically, and financially demanding.

In most cases, a high melting temperature of metals or ceramics has to be reached [7,8]. In other cases, compaction must be performed, or various chemical intermediates for the formation of porous preforms have to be used. The other area of interest is the development and improvement of foaming agents. TiH_2_ is the strongest foaming or blowing agent used to produce aluminum and magnesium alloy foams [9,10].

Partial sintering of the powder compact is the simplest method for the production of porous ceramic materials because it is performed at lower temperatures and for a shorter time than required for the fabrication of dense materials. The porosity is the percentage of the total volume of cavities between the powder particles. The porosity level can be controlled by optimizing the sintering process while the pore size is primarily determined by the initial grain size. Usually, the pore size is two to five times smaller than the average size of powder particles [11]. When compacted powders, composed of a mixture of metal and ceramic particles, are sintered, a problem arises from the hardness of the particles. It is because a high pressing power must be developed, and this highly contributes to wearing the pressing tools. Iron-based porous metals are considered an alternative to porous aluminum materials (foams) because steel has higher strength, higher energy absorption capacity, and is generally cheaper than aluminum. However, steel is denser than aluminum, so low-cost production remains a challenge because of its much higher melting point. A method of foaming cold-pressed precursors, which are made from pure powders of iron, graphite, and hematite at temperatures lower than a melting point, has been proposed [2]. This foaming of iron is driven by CO and CO_2_ which are generated by carbon-induced reducing iron oxide [12]. Among metallic materials, nickel has excellent corrosion resistance and is also resistant to salts and acids. These porous materials are commonly used to make functional materials such as filter materials, capillary wicks, and battery electrodes [13,14,15].

A pore-forming separating agent has also been used to prepare porous Ni skeletons [16]. For example, sodium chloride (NaCl) has been used when porous nickel materials were fabricated by sintering a mixture of nickel oxalate (NiC_2_O_4_) with NaCl. It has been shown that NaCl as a separator can effectively prevent any direct contact between nickel particles and thus reduce the aggregation of powder particles during the mixing. The porosity and the pore structure can be well controlled by varying the sintering temperature. Other agents for preparing porous materials with high porosity and controlled pore structure are NH_4_HCO_3_ [17,18,19], where the pores are formed by volatilization of reagent during sintering, or NaCl, where the porous nickel material was prepared by sintering nickel oxalate (NiC_2_O_4_) and NaCl after blending and reduction [20]. Hence, the main aim of our work is to design and validate the cost- and time-effective route for producing metal-ceramic porous skeletons.

The preparation of Ni-Al_2_O_3_ composite preforms with high open porosity for infiltration and filtration purposes was demonstrated in our previous paper [21]. The method was based on the oxidation of loose powder mixtures of Ni and Al_2_O_3_ with forming of oxide bonding on the powder interfaces. The loose powder has been used because these mixtures are too hard to be pressed and compacted to solid preforms. However, the loose powder can hardly obtain the desired complex preform shapes, and necessary compromises are to be met.

Currently, polymer plasticizer in Ni + Al_2_O_3_ mixture is usually used [22]. Then the mixture would be very tightly pressed, with very little rest porosity. As a result, the oxygen access to the inner volume would be suppressed, and only surface layers would be oxidized. The synthetic rubber, poly(ethylene glycol), or poly(vinyl alcohol) used as plasticizers are removed during the sintering at higher temperatures. Therefore, polymers do not affect the development of bonds between the particles. However, they could negatively affect the sintering process due to the ash residue and then cause the disintegration of tablets due to residual porosity [23].

In this work, the technology was improved by applying a metal Al binding agent to the Ni-Al_2_O_3_ mixtures that make the pressing feasible and provide the compact preforms with the desired open porosities and the possibility to shape the green compacts. Prepared powder mixtures were compacted using cold isostatic pressing (CIP) and then oxidized at 600 °C, 700 °C and 800 °C. Further, the microstructural and chemical analysis (SEM-EDS, XRD), TG/DTA measurements, hardness and thermal expansion measurements were performed to investigate the phase changes of the material.

## 2. Materials and Methods

### 2.1. Green Body Preparation from Ni + Al_2_O_3_ + Al Powder Mixture

The Ni powder (99.3%, Metco 56C-NS, OC Oerlikon Corporation, Pfäffikon, Switzerland) with a particle size of <75 µm, Al_2_O_3_ powder (95.50%, Metco Amdry 6060, OC Oerlikon Corporation, Pfäffikon, Switzerland) with a particle size of <45 µm and Al powder (95.50%, Alpoco A1050, AMG Alpoco UK Limited, UK) with a particle size of <63 µm were used for a green body preparation. The mixtures of Ni powder with 12.87 wt.% Al_2_O_3_ powder were prepared at first (i.e., 25 vol.% Al_2_O_3_). This composition was determined experimentally in our previous work because more than 12.87 wt. % Al_2_O_3_ resulted in a substantially weaker sample cohesion after thermal oxidation [21]. Because the pressing of hard powders is accompanied by difficulties, Al powder with relatively plastic particles was used as a binder. Therefore, 3 g of Al powder was further added to the mixture of Ni and Al_2_O_3_ powders. The Al amount was sufficient to ensure sample compactness but not at the expense of decreasing the sample porosity. The final powder mixture contained Ni 68.76 vol.%, Al_2_O_3_ 22.95 vol.%, and Al 8.29 vol.% (in the following text, it was stated as “powder mixture”). Al_2_O_3_ powder was sieved to obtain a fraction with a particle size of 20–32 µm. Analysis of the particle size distribution was performed for all used powders by laser diffraction in water using Analysette 22 NanoTec (Fritsch, Germany). The mean particle size (D50) value was 69.91 µm for the Ni powder, 33.17 µm for the Al_2_O_3_ powder, and 30.32 µm for the Al powder. Although the Al_2_O_3_ powder exhibited blocky shape morphologies, as stated by Oerlikon Metco company, laser diffraction provided reliable results because it has been shown that even for blocky particles, the D_50_ parameter is sufficiently accurate [24]. The particle morphology could also explain a slightly higher value of D_50_ 33.17 µm for the 20–32 µm Al_2_O_3_ fraction. Evidently, it is because narrow blocky-shaped particles with a length of more than 32 µm passed through a sieve with a mesh size of 32 µm. The powder mixture was vigorously blended in a mechanical blender, Turbula type T2F (WAB, Muttenz, Switzerland), for 30 min.

A green body compact was prepared from the powder mixture by cold isostatic pressing (CIP) using EPSI CIP 400-300*750 Y device (EPSI NV Walgoedstraat, Belgium). The pressing pressure was 370 MPa, and the final dimensions of the sample after CIP were 19 mm in diameter and 15 mm in length.

### 2.2. Differential Thermal Analysis and Thermogravimetric Measurement (DTA/TG)

**Figure 1:**
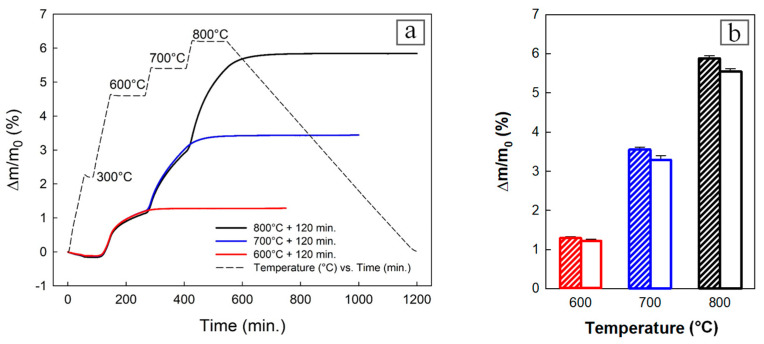
Weight gain of oxidized samples during heating in air. (**a**) Thermal oxidation was performed in the TG apparatus at the final temperature of 600 °C (red line), 700 °C (blue line), or 800 °C (black line). (**b**) Comparison of the final relative weight gain obtained for small samples subjected to TG analysis (hatched bars) and corresponding bulky CIP powder mixture oxidized in an oven (empty bars) at the final temperature of 600 °C (red), 700 °C (blue), or 800 °C (black).

Before oxidation, the sample with dimensions of 4 × 4 × 10 mm was cut off from the center of the green body compact prepared by CIP and put into a smaller cylindrical alumina crucible (height: 14 mm, diameter: 6 mm). This small sample was further subjected to thermal oxidation in the air to monitor the behavior of weight gain as a result of the NiO growth, Al melting, and Ni-Al reaction kinetics. Thermal oxidation was run in three different ways: 600 °C/120 min. dwell, 600 °C/120 min. dwell + 700 °C/120 min. dwell, and 600 °C/120 min. dwell, 700 °C/120 min. dwell and 800 °C/120 min. dwell at a heating rate of 5 °C/min (Figure 1a) in DTA/TG/Dil combined apparatus (Linseis Thermal Analyser L75/L81/2000, Selb, Germany). The spontaneous cooling rate was approximately 1.2 °C/min.

### 2.3. Thermal Oxidation

The CIP green body tablets with a diameter of 19 mm and a height of l0 mm were oxidized in a tubular oven, Type 018LP (Elektrické pece Svoboda, Světice u Říčan, Czech Republic) in static air. During heating, the temperature was gradually increased up to the final value of 600 °C, 700 °C or 800 °C and holding time for 120 min (Figure 1a). Before the final temperatures of 700 °C and 800°C were reached, the temperature in the oven was kept constant for 120 min at 600 °C, and 600 °C and 700 °C, respectively. The heating rate was 5 °C/min. The cooling was spontaneous with a rate of approximately 1.2 °C/min. The weight of the CIP green body tablets related to a newly formed NiO phase during the oxidation cycle was determined by the semi–micro balance R200D (Sartorius, Goettingen, Germany) with an accuracy of 0.1 mg.

### 2.4. Porosity Measurement

The open porosity of the sample was assessed by the Mercury Intrusion Porosimetry (MIP) method using Poremaster 60GT (Quantachrome Instruments, Boynton Beach, FL, USA) device with a maximum applied pressure of mercury at the level of 414 MPa.

### 2.5. Analyses of the Chemical Composition and Microstructure

The microstructure of the CIP green tablets before and after thermal oxidation at the final temperature of 600 °C, 700 °C, or 800 °C (Figure 1a) was determined by scanning electron microscopy (SEM) utilizing JEOL JSM 6610 (Jeol, Tokyo, Japan). The samples were cut into two identical halves and prepared metallographically. It is important to note that the samples were examined in the center of volume. A newly formed NiO and Ni-Al phases were identified and localized by combining SEM with energy dispersive X-ray spectroscopy (EDS, OI X-max 50 mm, Oxford Instruments, Tubney Woods, Abingdon, UK). The qualitative and semiquantitative phase compositions of the samples were studied by SEM-EDS and, more specifically, by X-ray diffraction (XRD) using Bruker AXS D8 (Bruker AXS GMBH, Karlsruhe, Germany) Advance diffractometer with Co Kα radiation in Bragg–Brentano configuration with 1D LYNXEYE linear detector. The collection time was effectively 368 s per step in steps of 0.04 deg. Semiquantitative phase analysis was carried out using a HighScore Plus 3.0 (Panalytical B. V., Almelo, The Netherlands); phase content determination accuracy was around 2 mass %.

### 2.6. Thermal Expansion

DTA/TG/Dil combined apparatus (Linseis Thermal Analyser L75/L81/2000, Selb, Germany) was used for monitoring thermal expansion. The sample temperature was measured by S-type thermocouple located in the close vicinity to the sample. The samples were heated from 30 °C to 800 °C at the heating rate of 3 °C/min in an inert argon atmosphere. All oxidized CIP preforms (600 °C, 700 °C, and 800 °C) were subjected to three heating/cooling cycles. Under the same experimental conditions, the correction measurements were performed using alumina standards. The calibration was performed according to DIN 51045 with NBS-Pt.

Rectangular specimens with a square base of 5 mm × 5 mm and a high of 10 mm were used for thermal expansion measurements. All these samples were cut off from the center of oxidized CIP compacts. Before measurement, the top and bottom surfaces of the samples were machined with a tolerance of ±0.02 mm.

### 2.7. Hardness Measurement

The hardness HV10 of the sample was determined by the hardness tester HPO-250KR/AQ (Aquastil Slovakia, Považská Bystrica, Slovakia) under the hardness load of 98.07 N applied for 10 s.

## 3. Results and Discussion

As the free inner space for oxygen access is predominantly crucial, Al powder was chosen as a binding agent. In this arrangement, alumina particles are stuck into plastic Al particles during CIP, and the entire tablet can be easily removed after compaction. Here, the diameter of Al particles is quite essential. Too fine particles might fill the interparticle spaces, and the subsequent infiltration would not be possible after compaction. Thus, the size and amount of Al particles can effectively regulate the porosity of the green body and improve the conditions for gas pressure infiltration with liquid metal [25,26], e.g., aluminum [27].

### 3.1. Kinetics of Thermal Oxidation of CIP-ed Powder Mixture

The CIP green tablets prepared from the Ni + Al_2_O_3_ + Al powder mixture were subjected to thermal oxidation. The three final temperatures were tested: 600 °C, 700 °C, and 800 °C to monitor the reaction kinetics of Ni-Al synthesis when Ni particles were covered with NiO layers. The heat produced during an exothermic reaction, when NiO is formed, will not equally dissipate from the tablet surface and its volume. To diminish the contribution of this event, gradual heating was utilized. In addition, keeping a constant temperature for 30 min. at 300 °C and 120 min. at 600 °C, 700 °C, and 800 °C promoted temperature balancing in the sample volume. When continual heating was used, the temperature inside the samples substantially increased, leading to the occurrence of sample cracks due to significant tension. Spontaneous cooling was slow in order to facilitate temperature balancing between a sample surface and volume. Time dependence of the sample’s relative weight gain was examined in the TG apparatus, where smaller samples were tested. Figure 1a documents that the relative weight gain reached during the heating–cooling cycle depends on the final heating temperature. When this increased from 600 °C to 800 °C, the relative weight gain increased by more than four-fold, from 1.313 ± 0.017% to 5.892 ± 0.062%. Because the volume of samples for TG analysis was almost four-fold smaller than that of CIP green tablets, which were appropriate for further testing, the relative weight gain between these two groups was compared. Figure 1b shows that smaller samples exhibited a slightly larger relative weight gain. This effect was more pronounced at the final heating temperatures of 700 °C and 800 °C. Obviously, it was because smaller samples were heated in entire volume more efficiently at a lower temperature.

**Figure 2:**
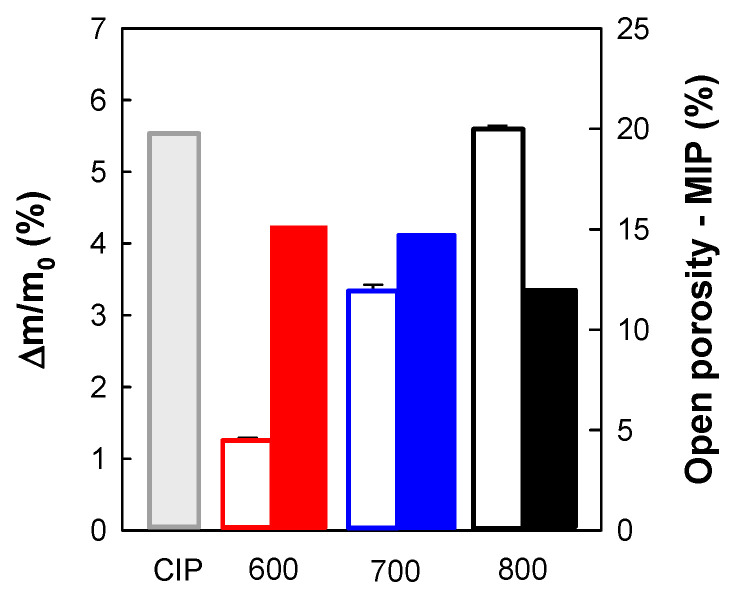
Correlation between the final relative weight gain and open porosity. CIP green compact was subjected to thermal oxidation at the final temperature of 600 °C (red), 700 °C (blue), or 800 °C (black). The final relative weight gain (empty bars) and open porosity (colored bars) were measured by MIP.

The weight gain in all three oxidized CIP green tablets was caused by NiO growing, as was already showed in our previous work [21]. The formation of NiO layers on the surface of Ni particles was associated with the lowering of sample porosity. Similarly, in our present study, the increasing weight gain correlates well with the decreasing open porosity, measured by the MIP method (Figure 2).

### 3.2. Microstructure and Chemical Analysis of the Green Body and Oxidized CIP-ed Powder Mixture Obtained by SEM-EDS Microscopy

**Figure 3:**
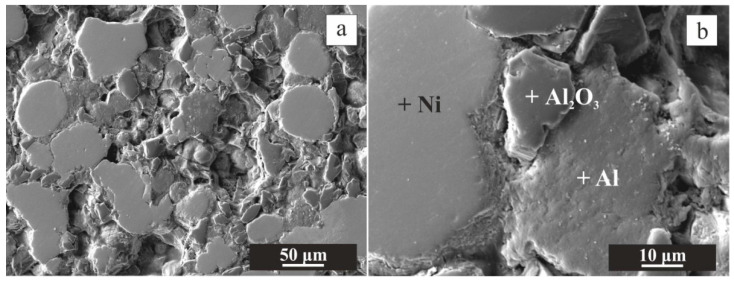
SEM images from the central region of the CIP green tablet at magnification 300× (**a**) and 1500× (**b**). The phases, determined by EDS, are denoted by cross (**b**).

During the pressing of the powder mixture by CIP, relatively plastic Al fulfilled the role of a binder agent and filled part of the empty spaces between hard Ni and Al_2_O_3_ powders. The coherence that is evident in Figure 3 was preserved.

**Figure 4:**
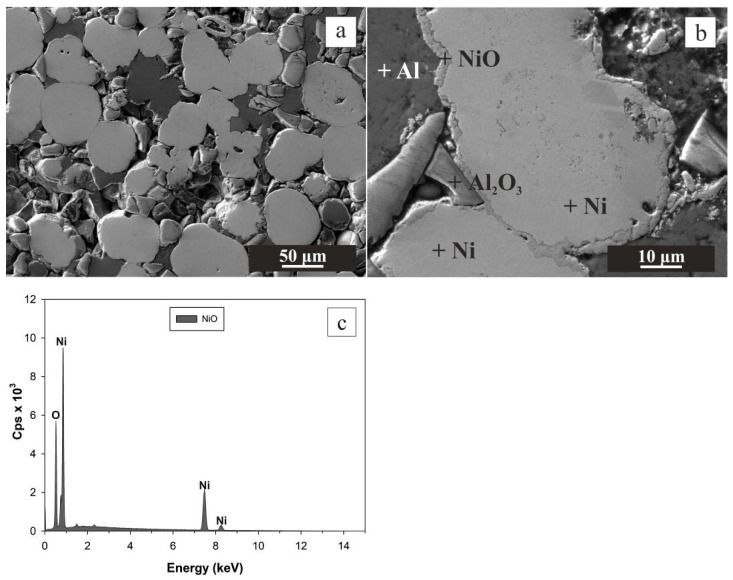
SEM images from the central region of the oxidized CIP green tablet at magnification 300× (**a**) and 1500× (**b**). Thermal oxidation was performed in an oven at the final temperature of 600 °C. The phases, determined by EDS, are denoted by the cross (**b**). EDS spectra were obtained by point analysis in NiO (**c**).

After oxidation to final temperature to 600 °C, the appearance of new NiO phase was confirmed by SEM EDS microstructure analysis (Figure 4). Open porosity of CIP green tablet before oxidation was 19.58 ± 0.00%. Thermal oxidation at the final temperature of 600 °C caused the increase in relative weight gain by 1.231 ± 0.035% that, in turn, resulted in only the slight decrease in open porosity by 4.34% to 15.24 ± 0.00% (Figure 2).

**Figure 5:**
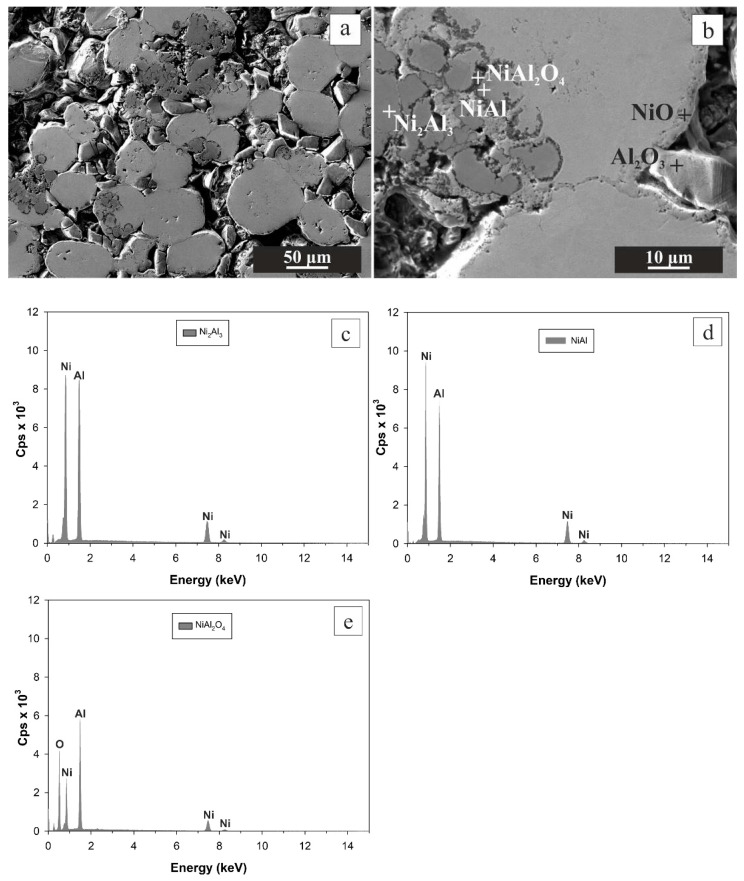
SEM images from the central region of the oxidized CIP green tablet at magnification 300× (**a**) and 1500× (**b**). Thermal oxidation was performed in an oven at the final temperature of 700 °C. The phases, determined by EDS, are denoted by the cross (**b**). EDS spectra were obtained by point analysis in Ni_2_Al_3_ (**c**), NiAl (**d**), and NiAl_2_O_4_ (**e**).

Increasing the final heating temperature from 600 °C to 700 °C resulted in a double increase in the relative weight gain to 3.311 ± 0.089%, reflecting the increase in the NiO phase, which was associated with a substantial decrease in open porosity measured by MIP to 14.59 ± 0.03% (Figure 2). SEM-EDS and XRD analyses identified three new phases in addition to NiO namely Ni_2_Al_3_, NiAl, and NiAl_2_O_4_ (Figure 5). The larger amount of the Al has been consumed to form Ni aluminides and NiAl_2_O_4_ (spinel).

**Figure 6:**
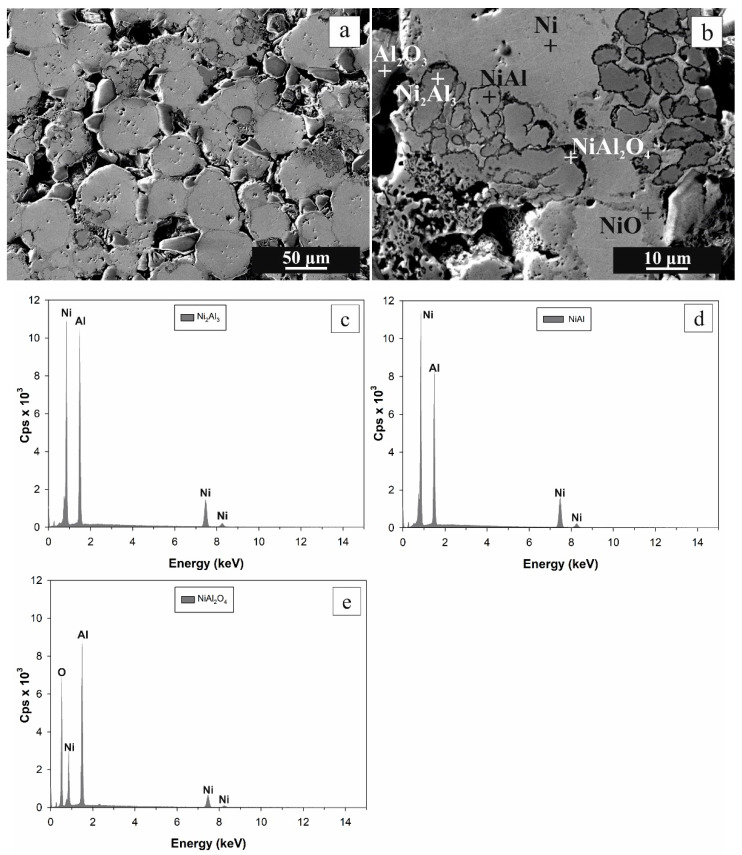
SEM images from the central region of the oxidized CIP green tablet at magnification 300× (**a**) and 1500× (**b**). Thermal oxidation was performed in an oven at the final temperature of 800 °C. The phases, determined by EDS, are denoted by the cross (**b**). EDS spectra were obtained by point analysis in Ni_2_Al_3_ (**c**), NiAl (**d**), and NiAl_2_O_4_ (**e**).

**Figure 7:**
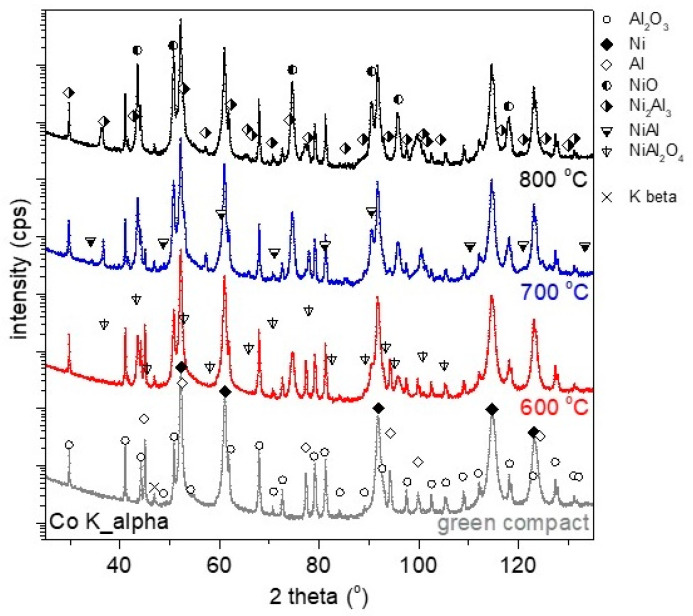
X-ray diffraction of the green compact (dark gray color), oxidized compact to 600 °C + 2 h hold (red color), oxidized compact to 700°C + 2 h hold (blue color), and oxidized compact to 800 °C + 2 h hold (black color), data shifted vertically for clarity.

When the final heating temperature further grew from 700 °C to 800 °C (Figure 6), the relative weight gain was increased up to 5.568 ± 0.051 %, and open porosity was decreased to 11.87 ± 8 × 10³ % (Figure 2). Concerning chemical composition, neither new phase was generated (Figure 7).

### 3.3. Chemical Analysis of the Green Body and Oxidized CIP-ed Powder Mixture Obtained by XRD Analysis

**Figure 8:**
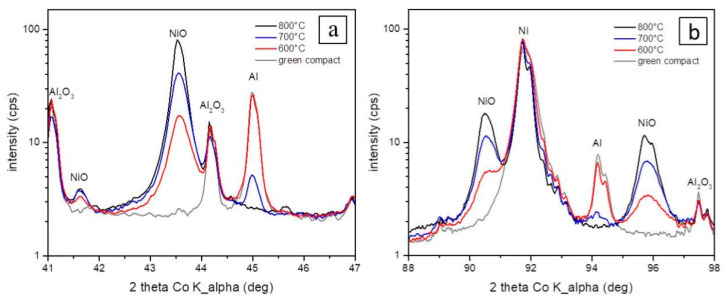
Selected parts of the X-ray diffraction patterns shown in Figure 8 show the evolution of NiO (**a**), Ni, and Al and the Al_2_O_3_ (**b**) stability with processing temperature.

Due to the rather large grain/particle sizes of the constituent powders used for compact green preparation (typically above 50 µm as described in Section 2.1) and their elemental composition, which leads to micro absorption effects, the use of quantitative or semiquantitative XRD analysis of phase content would be improper and would lead to significant errors. For this reason, only a qualitative comparison of the evolution of phase content is shown (Figure 7 and Figure 8). It is worthwhile to comment that the green compact, as well as the corresponding precursor powder mixture prior to compaction, were subjected to micronizing in an attempt to minimize the above-mentioned effects using the McCrone micronizing mill and rotation of samples during XRD measurements. Semiquantitative analysis of obtained XRD data (not shown) has not led to a good match of the phase content against nominal values (accuracy not better than 5%), indicating that probably micro absorption and porosity are still significant issues.

A comparison of the XRD diffractograms (Figure 7) revealed that the content of the NiO phase increased with increasing temperature and time. Intermetallic phases Ni_2_Al_3_, NiAl, and NiAl_2_O_4_ are observed only in samples oxidized to 700 °C and 800 °C (blue and black pattern). It is further shown that the formation of these phases is accompanied by the loss of Al (Figure 7 and Figure 8a,b). As stated above, using SEM-EDS analysis, it was problematic to find pure Al at these temperatures. However, by the XRD method, pure Al was detected in the sample oxidized at 700 °C (Figure 8a—blue pattern), which means that at this temperature, there was no complete consumption of Al for aluminides or spinel, respectively. At 800 °C, pure Al was no longer detected even by the XRD method (Figure 8a—black pattern), which means that it had been completely transformed into aluminides or spinel.

### 3.4. Differential Thermal Analysis of Green Body Powder Mixture

The effect of the atmosphere on the processes leading to perform formation was studied via DTA measurements. These were performed both in an argon atmosphere and in the air up to the temperature of 800 °C.

**Figure 9:**
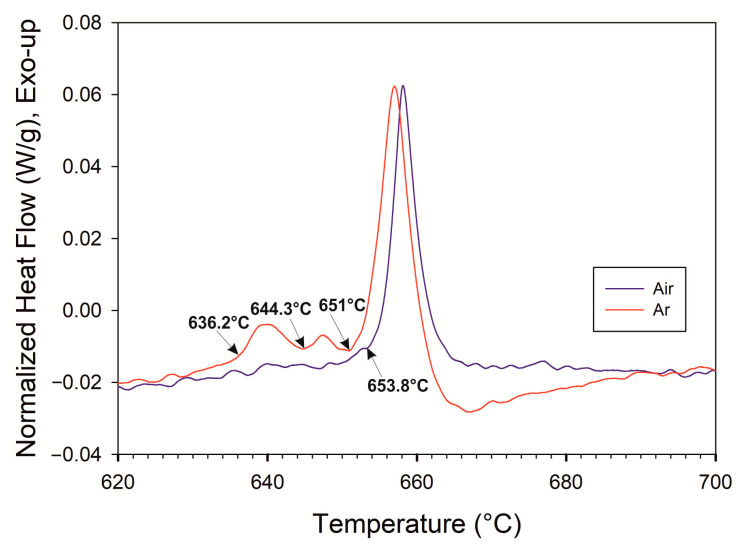
DTA measurement of CIP-ed green compact in air and in argon.

The Ni + Al_2_O_3_ + Al powder green compacts subjected to the DTA measurement in argon exhibited several exothermic reactions upon heating with the onset at 636.2 °C, 644.3 °C, and 651.3 °C as shown in Figure 9. These reactions can be related to the formation of various Ni aluminides (NiAl_3_, Ni_2_Al_3_, NiAl) [28], NiAl_2_O_4_ spinel and melting of the rest of Al. However, the sequence and extent of reactions are in this powder arrangement ruled by the local chemistry. The crucial result of this measurement is that due to the reactions and accompanying volumetric changes, the integrity of the green compact was fully destroyed and the sample broke into several pieces.

The DTA measurement performed in air recorded only one exothermic reaction with the onset of 653.8 °C, i.e., close to the melting temperature of Al. The important result is that the sample showed no signs of disintegration and could have been easily removed from the crucible. With respect to the microstructural observations, this can be ascribed to the air-stimulated formation of NiO reinforcing and supporting the internal architecture of the preform.

These experiments undoubtedly confirmed the decisive role of the atmosphere on the stability of the Ni + Al_2_O_3_ + Al preform. A similar result was achieved in our previous report [29], however, with only Ni-Al green compacts.

**Figure 10:**
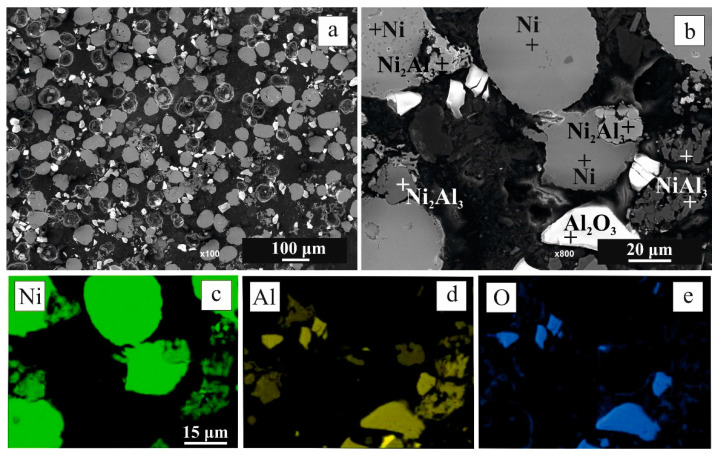
SEM-EDS images of the disintegrated crack mixture after annealing in Argon; macrostructure (**a**), corresponding detail in higher magnification with point elemental composition (**b**), and EDS elemental mapping, i.e., Ni (**c**), Al (**d**) and O (**e**).

SEM-EDS images (Figure 10) of broken green CIP compact after heating in argon correspond to the exo-reactions-red curve in Figure 9. They indicate that aluminide does not break itself to disintegrate the compact. More probably, after their formation, aluminides break away from the nickel particle in argon. After separation from Ni, diffusion of Al to Ni particle is not possible. The observed aluminides are primarily Ni_2_Al_3_. A change in the density can cause this detachment (see data in Table 1) due to the different crystalline structures of aluminide vs. Ni particles and/or the different CTE of aluminide vs. Ni particles. As expected, no NiO was revealed.

This is another reason why it is necessary to oxidize Ni particles at 600 °C and above since the proper formation of NiO, together with the formation of aluminides, leads to the creation of spinels that hold the materials together and enable further manipulations [39,40].

### 3.5. Thermal Stability of Oxidized CIP-ed Powder Mixture

To study the thermal stability of oxidized samples, they were subjected to thermal cycling in an inert argon atmosphere up to 800 °C. The thermal expansion and coefficient of thermal expansion (CTE) were measured during this. Three dilatometric measurements were performed for each sample to monitor the thermal stability. The course of individual thermal expansion curves was recorded and compared.

**Figure 11:**
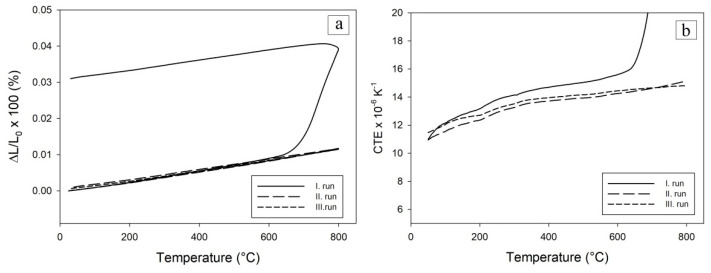
Relative elongation (**a**) and coefficient of the thermal expansion (**b**) were observed for the green compact oxidized to 600 °C and subsequently thermocycling to 800 °C in the Ar atmosphere.

The least thermally and also structurally stable was the green body compact sample oxidized to 600 °C (Figure 11). Its permanent extension was 3.3%. (Figure 11a) after the first cycle. During the first cycle, this sample began to expand significantly at a temperature of 671.3 °C, which is also shown on the CTE record (Figure 11b). This high increase in length could be attributed to the reaction of the unreacted aluminum in the sample (see XRD at Figure 8a) reacting with Ni, despite the fact that a protective layer of NiO was formed on the Ni particles after oxidation. The second and third cycles exhibited no further permanent deformations confirming that the transformation of the rests of Al has been mostly completed during the first cycle.

**Figure 12:**
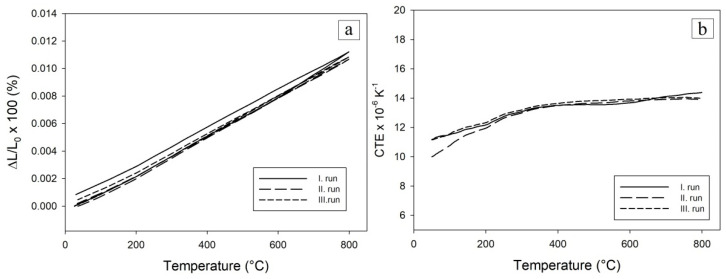
Relative elongation (**a**) and coefficient of the thermal expansion (**b**) were observed for the green compact oxidized to 700 °C and subsequently thermocycled to 800 °C in the Ar atmosphere.

Green body compact oxidized at the temperature of 700 °C had a permanent elongation of 0.07% after the first cycle (Figure 12). This can be attributed to the small amount of pure unreacted Al in an oxidized sample as confirmed by X-ray measurements. Similarly, to the previous sample, it reacted in the first cycle with Ni, but due to the limited amount of Al, the permanent elongation was much smaller.

**Figure 13:**
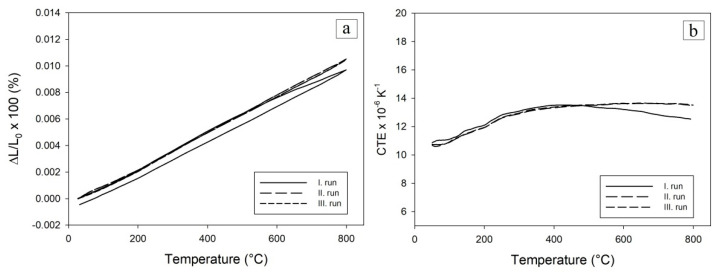
Relative elongation (**a**) and coefficient of the thermal expansion (**b**) were observed for the green body compact oxidized to 800 °C and subsequently thermocycled to 800 °C in the Ar atmosphere.

Green body compact oxidized at a temperature of 800 °C exhibited negative permanent elongation, i.e., it shortened by 0.06% (Figure 13). There was no rest Al in the structure obviously responsible for the thermal expansion in previous cases. This structure showed the best structural stability. However, further evolution of equilibrium aluminides and sintering processes manifested by the decrease of overall open porosity (Figure 2) took place during the applied thermal cycling. Thermal expansions in the second and third cycles are identical.

During the thermocycling of samples, it was also observed that samples oxidized at 600 °C and 700 °C had a slightly increased CTE after the third measurement cycle as after the second. The sample oxidized at 800 °C had identical CTEs in the course of second and third cycle.

As it is evident from DTA measurement that after CTE runs, there could be slight microstructural changes and also the release of internal stresses, therefore, the porosity measurements by MIP were repeated after the third CTE runs in argon (Table 2).

**Figure 14:**
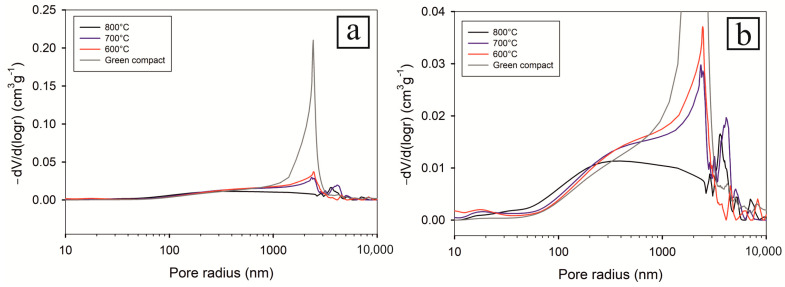
Pore size distribution after oxidation at 600 °C, 700 °C and 800 °C in comparison with non-oxidized green compact: whole range (**a**), detail (**b**).

**Figure 15:**
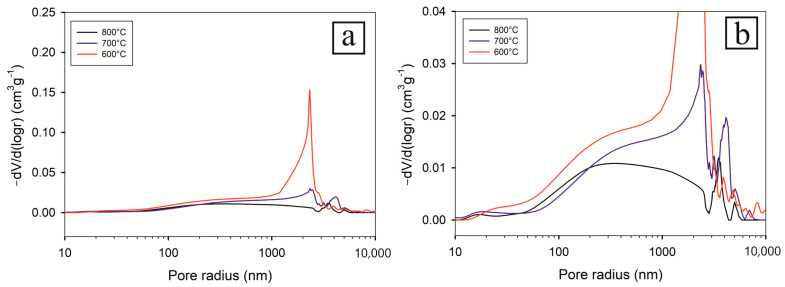
Pore size distribution of oxidized samples after thermocycling at 800 °C in argon: whole range (**a**), detail (**b**).

The data obtained from MIP (Figure 14 and Figure 15) measurements correspond well with those from dilatometry. It is clearly evident that with increasing oxidation, the porosity gradually decreases. Consistent with the previous observations, the ratio of pores with a radius larger than 1 µm with increasing temperature decreases, whereas the number of pores smaller than 0.5 µm increases. The increased number of smaller pores is also visible on SEM microstructures (Figure 4, Figure 5 and Figure 6).

The general trend is the decrease of porosity after oxidation and thermocycling in Ar. It decreased from 19.58% down to 11.16% after oxidation at 800 °C and thermocycling at 800 °C in Ar. However, after oxidation at 600 °C, the thermocycling at 800 °C in Ar led surprisingly to a porosity increase up to 20.02%, even above the green body porosity of 19.58%. Moreover, the error of the MIP open porosity measurements increased significantly after thermocycling (Table 2). It can be attributed to the change in the open pore system of the samples due to volumetric changes and internal stress relaxations after the first runs of thermocycling.

**Figure 16:**
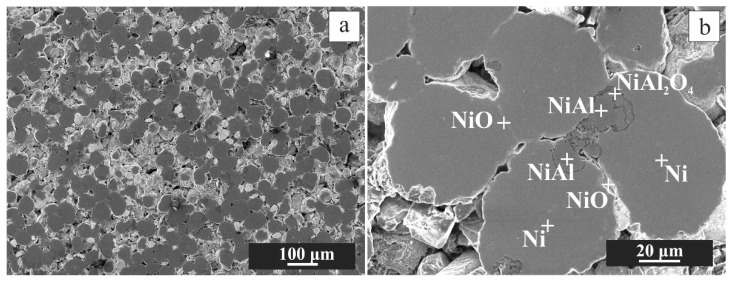
SEM macrostructure of the sample oxidizes at 600 °C after thermocycling in Ar (**a**), with the phases determined by EDS, are denoted by a cross (**b**).

This corresponds perfectly with the thermal expansion measurements and the dramatic increase of elongation in the first thermal cycle. The compact oxidized at 600 °C contained unreacted Al and a relatively weak NiO supporting layer. During thermocycling in Ar (up to 800 °C), Al reacted with Ni, which resulted in the development of new phases (Figure 16) and corresponding volumetric changes. This was accompanied by the porosity increase.

As there were no significant dimensional changes after the second and third runs (Figure 11), it appears that the MIP measurements reflect the porosity after the first run.

During oxidations at 700 °C and 800 °C, already formed aluminides are connected with a strong NiO layer so that during thermocycling, there were only slight volumetric changes of the samples containing NiO + NiAl spinel + aluminide.

### 3.6. Hardness Measurement

**Figure 17:**
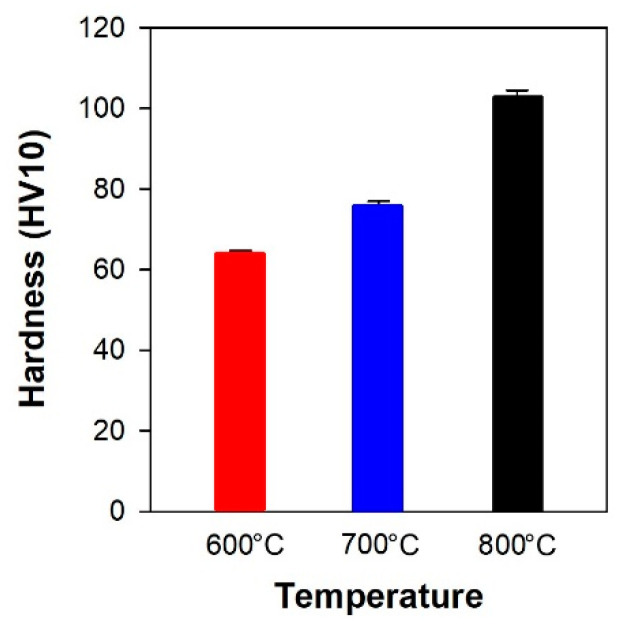
Comparison of the hardness for samples after oxidation up to 600 °C, 700 °C and 800 °C.

HV 10 hardness was measured on the samples. The sample oxidized up to 600 °C had the smallest hardness value of 64 ± 0.62, the sample oxidized up to 700 °C had a hardness of 75.7 ± 1.29, and the sample oxidized up to 800 °C had the hardest value of 102.9 ± 1.56 (Figure 17).

The observed increase can be explained on the basis of microstructures as follows: The sample oxidized up to 600 °C still contains pure non-reacted Al, and the NiO layer is the thinnest; therefore, it had the lowest hardness. The sample oxidized up to 700 °C still contained a small amount of non-reacted Al in the volume, but the NiO layer is thicker, and there are also formed Ni aluminides. The sample oxidized up to 800 °C already had completely reacted Al into aluminides, and the thickness of the NiO layer is the largest. In general, however, the thickness variations of NiO and the formation of in-situ Ni aluminides, where both NiO and Ni aluminides form a heterogeneous binder, have a significant effect on the hardness of the material. The hardness increases with increasing oxidation temperature.

## 4. Conclusions

Powder mixtures of relatively hard materials Ni and + 25 vol.% Al_2_O_3_ with the addition of Al powder was compacted using the CIP method at a pressure of 370 MPa and oxidised at temperatures of 600 °C, 700 °C, and 800 °C. To achieve the desired consistency, Al powder was added as a binding agent, and the green mixtures had a ratio of Ni 68.76 vol.%, Al_2_O_3_ 22.95 vol.%, and Al 8.29 vol.%. The following results were obtained:

- The oxidation of green compacts to temperatures of 600 °C, 700 °C, and 800 °C has led to the decrease of porosity from 19.58 ± 0.00% (as-CIPed) to 15.24 ± 0.00% (600 °C), 14.59 ± 0.03% (700 °C) and 11.87 ± 8 × 10³ % (800 °C), respectively.
- The microstructural analysis confirmed the formation of thin NiO after oxidation at 600 °C, the formation of a coarse NiO layer, as well as the formation of aluminides Ni_2_Al_3_, NiAl, and spinel NiAl_2_O_4_ after oxidation at 700 °C and 800 °C. XRD measurements confirmed that these reactions were triggered by Al reacting with Ni.
- These reactions were monitored by DTA measurements. Measurements performed in Ar revealed the full disintegration of samples suffering from the lack of stability due to missing NiO support. The sample remained consistent after the DTA measurement in the air confirming thus the influence of the atmosphere on the stability of the final preform.
- The thermal expansion measurements confirmed the dimensional stability of oxidized samples after oxidation at 700 °C or 800 °C, i.e., after the completion of exothermic reactions of Al with Ni.
- The samples oxidized up to 600 °C, 700 °C and 800 °C have the hardness value of 64 ± 0.62, 75.7 ± 1.29, and 102.9 ± 1.56, respectively. The hardness increases with increasing oxidation temperature due to the thickness variations of NiO and the formation of in-situ Ni aluminides from Al binding agent. Both NiO and Ni aluminides have a significant effect on the hardness of the prepared material.

It can be concluded that the modification of the preform porosity for gas pressure infiltration is also possible in other ways than the variation of the size of the Al_2_O_3_ particles.

## Figures and Tables

**Table 1:** Density of raw materials and observed phases.

	Ni	Al	Al_2_O_3_	NiO	NiAl_3_	Ni_2_Al_3_	NiAl	NiAl_2_O_4_
Density (g/cm^3^)	8.9 [30,31]	2.7 [32]	3.90–3.97 [33]	5.74–6.8 [34]	7.5 [35]	4.76 [36]	5.96 [37]	3.44 [38]

**Table 2:** Evolution of the green compact porosity after oxidation at 600 °C, 700 °C, and 800 °C and after subsequent thermocycling at 800 °C in an inert argon atmosphere.

Porosity of the green compact	19.58 ± 0.00%		
Oxidation regime (Figure 1a)	600 °C	700 °C	800 °C
Porosity after oxidation (Figure 1b)	15.24 ± 0.00%	14.59 ± 0.03%	11.87 ± 8 × 10³%
Porosity after thermocycling at 800 °C in Ar	20.02 ± 0.43%	14.30 ± 0.12%	11.16 ± 0.11%

## Data Availability

Not applicable.
